# Home Learning Environment and Screen Time Differentially Mediate the Relationship Between Socioeconomic Status and Preschoolers’ Learning and Behavioural Profiles

**DOI:** 10.1007/s10578-024-01724-z

**Published:** 2024-06-13

**Authors:** Paola Bonifacci, Diego Compiani, Chiara Vassura, Alexandra Affranti, Benedetta Peri, Viola Ravaldini, Valentina Tobia

**Affiliations:** 1 Department of Psychology, University of Bologna, Viale Berti Pichat 5, 40127 Bologna, Italy; 2 Faculty of Psychology, University Vita-Salute San Raffaele, Via Olgettina 58, 20132 Milan, Italy; 3 IRCCS San Raffaele Hospital – Ville Turro, Via Stamira d’Ancona 20, 20127 Milan, Italy

**Keywords:** Screen time, Home literacy, Home numeracy, Socioeconomic status, Behavioural problems

## Abstract

Environmental variables related to the home context, including home literacy and numeracy, screen exposure and Socioeconomic Status (SES) are potential risks or protective factors for children’s academic achievements and behaviour. The present multi-informant study aims to contribute to this issue by investigating SES’s direct and indirect relationships in early learning (i.e., literacy, numeracy, and cognitive) and behavioural skills within a large sample of young children. One parent and one teacher for each of 1660 preschoolers filled out a questionnaire investigating SES, tablet and TV use, home learning activities, behavioural problems/strengths (parents’ questionnaire), and children’s learning skills and behaviour (teachers’ questionnaire). Results of path analysis showed that tablet time and home learning environment mediate the effect of SES on early learning as assessed by teachers; as for the home learning environment, it was also a mediator of the relationship between SES and behavioural problems. Implications of these results for research in the field and educational policies are discussed.

## Introduction

The role of environmental factors in preschool years on children’s cognitive and academic skills has received increasing attention. It stands within previous theoretical models that highlighted the important role of environmental factors in development, such as Lewin’s behavioural equation [1], the bioecological model [2], and neuroconstructivism [3].

The Home Learning Environment (HLE) has been defined [4] as a multi-componential system that includes three main dimensions: structural characteristics (e.g., socioeconomic status—SES), educational beliefs (e.g., parents’ education values and beliefs), and educational processes (e.g., literacy and numeracy activities). These dimensions can be clustered into domain-general (e.g., SES, emotional support) and domain-specific (literacy and numeracy) processes. Other authors [5, 6] also include parental attributes (e.g., motivation, mental health, skills and knowledge) and the emotional climate of the parent–child relationship, as well as cultural settings. All these factors might contribute to shaping a child’s achievement through reciprocal interactions between the child’s individual traits and the HLE.

Nowadays, many children spend varying amounts of time engaging with electronic devices such as televisions, tablets and smartphones. Previous literature has reported that higher screen exposure times might be related to possible adverse outcomes in children’s achievements and behaviour, particularly for the youngest (for meta-analysis, see [7]). However, there is also evidence of positive effects for specific contents (e.g., digital book reading [8], prosocial games [9]), suggesting that some tools might motivate children in learning opportunities [10]. Therefore, digital device use has started to enter into HLE models [11, 12].

The present study focuses on SES, home literacy and numeracy practices, screen daily exposure as concurrent predictors of children’s early learning (i.e., literacy numeracy and cognitive skills) and behavioural outcomes, adopting a multi-informant approach including teachers’ and parents’ reports.

### Early Literacy and Numeracy Skills and their Relationships with the Home Learning Environment

During preschool years, through implicit and explicit learning processes, children develop basic math skills, such as counting, magnitude comparison, number knowledge [13, 14], and pre-literacy skills, usually including letter knowledge, phonemic awareness, rapid automatized naming, and verbal knowledge [15, 16]; see [17] for Italian. These early numeracy and literacy skills are related to the further development of, respectively, complex mathematical abilities [18–21] and mature literacy skills (decoding and reading comprehension) [22, 23], which are taught at school. Early literacy and numeracy skills can be either evaluated by using objective tasks or through teachers’ reports, the latter result in reliable tools [24, 25]. In particular, teachers’ reports seem more strongly associated with children’s skills when considering cognitive variables [26].

The efficiency of early literacy and numeracy skills is modelled by the type and quality of activities that parents adopt in the home environment. Home literacy [27] and numeracy [28] activities have been proven to be related to the development of children’s academic skills (for meta-analyses, see, e.g., [29, 30]).

Previous studies have found positive associations between home literacy activities and literacy development [31–35], although diverse effect sizes and patterns of moderators have been reported [36]. Similarly, there is evidence of a relationship between home numeracy and numerical development [24, 37–42]. The home literacy and the home numeracy environment are cohesive parts of a global HLE [43], which was also found to predict higher secondary school tracks recommended by teachers at the end of primary school [44]. Most of the literature on home literacy and numeracy was obtained using parents’ self-report questionnaires, which showed reliable results [34, 38], suggesting that parents’ reports can be considered suitable tools in this research field [45].

Although many studies report a significant positive relationship between home literacy and numeracy environment and children’s early skills, there are heterogeneities in existing findings (e.g., [25, 29, 30, 46]) that might be due, at least in part, to the involvement of other (possibly confounding) factors that may affect the home environment, including family SES, as well as other everyday habits, such as exposure to screens.

### The Role of Socioeconomic Status on Children’s Early Literacy, Numeracy, and Behavioural Skills

Variation in socioeconomic status (SES), defined as “the social standing or class of an individual or group” [47], has been found to relate to augmented risks of underachievement in low-SES populations, e.g. [48, 49], although preschool program attendance might reduce the impact of socio-economic disadvantage [50].

Previous studies reported a negative impact of low SES on vocabulary knowledge and vocabulary growth [51, 52], early literacy [53], early numeracy [54, 55], and listening/reading comprehension skills [56, 57]. Also, disparities in children’s well-being and mental health concerning SES emerged [58]. Multiple mechanisms might explain these relationships. Children (and families) from low socioeconomic backgrounds have less access to material and social resources to support cognitive and emotional development, which is linked to the development of psychopathology and lower academic achievement [48]. SES levels might influence cognitive and literacy development, mediating the educational opportunities that can be achieved (e.g., exposure to books, reading practice, quality of schools, etc.) with possible long-term outcomes [59]. Additionally, socioeconomic pressures may negatively affect parent–child relationships, which have a knock-on effect on development [60].

However, the strength of the relationship between SES and measures of academic achievement and psychological well-being is under debate. Contrasting results are reported in the literature, and many factors have been suggested to mediate the relationship between SES and children’s outcome measures. For example, recent meta-analyses found diminished, despite being significant, effect sizes of the relationship between SES and academic achievement [61–63] than those suggested by previous literature [64] and the effect size was more robust in more economically developed countries [65].

Also, the relationship between SES and academic achievement is more robust in younger children but tends to decrease in adolescents (see [55] for results on math skills). Similarly, regarding the relationship between SES and mental health, results suggest a weaker effect size in older compared to younger children [66] and a variation in different populations and communities [58].

In addition, the impact of SES might vary based on the type of outcome considered. For example, in the math domain, SES disparities are differently related to subcomponents of numeracy skills (for a review, see [67]). In the psychopathology domain, SES has been found to relate more to externalising than internalising disorders [58].

Specifically, SES might relate to children’s skills through home learning environment practices. Previous studies have shown that parental involvement at home is unequally distributed by SES [48, 68], and [69] reported later parental involvement in literacy activities in low-SES families. However, the associations between SES and home literacy are typically moderate in magnitude [70–73]. This suggests high variability within both low- and high-SES families in their support for home learning. Furthermore, some studies suggest that home activities may serve as a buffer promoting resilience in low SES context [74]. In the numeracy domain, some studies found a positive association between home numeracy and SES [75–77], whereas others found the opposite [78] or no relationship [28, 79].

A study on Italian preschoolers [53] found that home literacy partially mediates the effect of SES on language, literacy, and non-symbolic numeracy measures and fully mediates the relationship between SES and symbolic numeracy skills, suggesting that an enhanced home learning environment might mediate the role of SES on children’s skills. Similarly, [77] found that home numeracy mediates the relationship between SES and children’s numeracy skills. HLE was also found to mediate the relationship between SES and ADHD symptoms [80]. These results align with the idea that home experiences might be viewed as proximal variables directly related to children’s outcomes, whereas SES should be considered as a distal variable [81].

Finally, it is essential to underline that SES is better viewed as a structural variable related to the family’s circumstances. Therefore, the disadvantage is not an inherent characteristic of the family or the individual themselves but should be viewed within the relationships between individuals, society, and the school system [82]. From this perspective, the impact of SES should not be considered from a deterministic perspective; rather, it can be modulated by other social and family characteristics. Also, results might vary according to how SES is measured. Although many indices can be considered, parental education and income/occupation are usually the most frequently used.

### The Impact of Screen Exposure on Children’s Cognitive and Behavioural Skills

Recent investigations showed that, over the past 20 years, the daily duration of screen time (i.e., time spent with screen-based devices, such as television, tablets and smartphones) that young children are exposed to is increasing, while age of first exposure is decreasing [83]. In addition, a large amount of scientific literature has highlighted the potential detrimental effects of screen-based devices, especially on children as young as preschoolers. Notably, adverse effects on physical health (e.g., obesity and short sleep duration) [7], cognitive and linguistic skills [84, 85], behavioural (e.g., internalising and externalising symptoms) and social traits (e.g., peer relationships) [86], as well as attentional problems [87] have been reported. However, positive effects of screen-based devices have also been shown (e.g., improved learning using mobile applications [88]). There is evidence that high-quality learning apps may support children’s early literacy (e.g., print and sound knowledge [89]), cognitive and even behavioural competencies [8, 9]). Finally, other studies have not found significant relationships between screen time and cognitive or behavioural variables (e.g., [90]).

In the present work, we directly compared the relationships between “passive” television watching and “active” tablet use with young children’s early learning (i.e., literacy, numeracy, and cognitive) and behaviour. The distinction between "passive” and “active” was made based on the interactive nature of tablet devices compared to the more passive consumption typically associated with traditional television viewing [91, 92]. Among the few studies that have investigated this issue, a recent review [84] has examined the associations between media use and children’s (aged 0 to 5) cognitive skills, showing that, despite experimental research suggesting that the interactivity allowed by mobile devices has benefits over passive viewing (e.g., television) for learning, studies in naturalistic contexts have revealed that increased use of mobile devices is associated with poorer language and self-regulation.

Some studies compared the use of TV and tablets with behavioural strengths and difficulties. For example, [93] investigated the longitudinal relationship between program viewing and electronic media use and the Strengths and Difficulties Questionnaire (SDQ; [94]), teacher version, and attentional tasks administered 1 year later in a sample of preschoolers. Both types of habits investigated showed negative relationships, with higher levels of program viewing predicting an increase in externalising behaviours and total difficulties (SDQ) and children using apps more than 30 min per day showing a significantly lower inhibition score compared to low-dose (< 30 min) app users.

Another issue is the interference that children’s use of television or tablets could have on verbal and nonverbal interactions with their parents. Research shows poorer interactions associated with more extensive use of television (e.g., [95]) or tablets (e.g., [96]), basically due to the role of these media as “audiovisual distractors” that alter parent–child interactions in family homes [97]. However, the context in which the media are used (e.g., spending screen time alone or with a parent) could modulate the effects of parent–child interactions and developmental outcomes [96].

Some studies included the frequency of digital media use in HLE models. For example, [11] reported that digital and traditional HL activities were more related in the toddler group compared to the preschool sample. Also, different paths of relationships were found with children’s academic and socio-emotional outcomes measured through parents’ reports. Within the toddler group, a positive relationship between analogue HLE and socio-emotional and practical life skills was observed. However, for those who had lower exposure to analogue HLE, increased exposure to digital HLE was a moderator for greater socio-emotional skills. As for the preschooler age group, digital HLE activities were associated with weaker self-reported socio-emotional skills. Finally, both the digital and analogue HLE were positively associated with academic skills, although the analogue HLE showed higher effect sizes. Another study on children with migration background by [12] found that the association between migration background and children’s early linguistic abilities was mediated positively by the HLE and negatively by television exposure.

Finally, there is evidence that screen exposure can be related to SES; lower socioeconomic positions are associated with a cumulative increase in the time spent on screen-based entertainment [98].

To sum up, the home environment contributes to behavioural and cognitive development and different dimensions should be considered, including SES, home literacy/numeracy, and screen exposure. These factors are not independent but are strictly intertwined. Therefore, it is crucial to develop models that include different dimensions of the home environment and a multi-informant approach to children’s abilities and behaviour.

### Present Study

The main aim of the present study is to investigate the relationships between SES, home literacy/numeracy, and exposure to screens and children’s early literacy and numeracy skills as well as their emotional and behavioural profile.

The main research questions are the following:

1) Does SES concurrently predict home environment practices, children’s early learning skills, and behavioural profile?

Based on previous findings, higher levels of SES are expected to be positively related to home literacy (e.g., [69]) and numeracy practices (e.g., [76]), and negatively related to screen exposure [98]. Similarly, higher SES is expected to be related to better early literacy (e.g., [53]) and numeracy (e.g., [54]) skills. On the counterpart, lower SES levels should be related to greater emotional and behavioural problems in children, mainly regarding externalising symptoms (e.g., [58]). However, given the heterogeneity in previous results, the present study aims to evaluate the strengths of these relationships within a model where all the above-cited factors are included.

2) Do home learning activities and screen exposure (tablets and television) concurrently predict children’s early literacy/numeracy skills and behavioural profile?

Based on previous research, a positive relationship is expected between home literacy and numeracy practices and teachers’ evaluation of children’s early literacy and numeracy skills (see [29, 30]). Similarly, higher exposure to screens is expected to be related to weaker early literacy and numeracy skills (e.g., [85]) and increased emotional and behavioural problems (e.g., [93]). In addition to previous studies, the present study compares, within a unique model, the associations between HLE (screen exposure and home literacy and numeracy) and children’s early learning skills and behavioural profile, thus allowing us to explore which factor represents the best concurrent predictor.

3) Does the home environment mediate the relationship between SES and children’s skills?

Previous literature has highlighted the heterogeneity of results in the relationship between SES and children’s skills and behavioural profiles and has suggested that the home learning environment could act as a mediator [53, 77]. The present study adds screen exposure as a candidate mediator, and mediation effects of the home environment are expected in the relation between SES and children’s profiles.

## Method

### Participants

A community sample of 1660 children (48.6% females, mean age = 5.28 ± 0.61 years old) attending the 2nd and 3rd year of 58 public all-day preschools was involved in the study. The schools were located in areas with varying socio-economic statuses in the Municipality of Bologna (Italy). 28,6% of the participants had at least one parent who speaks a language different from Italian at home. A small portion (1.1%) of the sample (n = 17) had a certified mild disability. For 1660 children, parents filled out the study questionnaire (see below for details). Then, a teacher filled out a questionnaire for each child, for a total of 97 teachers involved.

In the Italian schooling system, preschool is attended by children aged 3 to 6 years old (a 3-year program), and no formal teaching of literacy or math is provided. However, the children might be engaged in activities to improve numeracy and literacy skills carried out in playful activities.

## Instruments

### Home Learning Environment

*Socio-Economic Status (SES).* Parents completed the Four Factor Index of Social Status (SES, [99]). To achieve a composite score for each child’s SES, information regarding parents’ educational level and occupation was scored from 1 to 7 for educational level and 1 to 9 for occupation. Then, SES scores for each parent were calculated using the formula (educational level*3 + occupation*5); the mean between parents’ SES was used as the child’s SES. The minimum and maximum scores ranged from 8 to 66.

### Home Literacy /Numeracy and Screen Exposure

The questionnaire included four questions developed for the present study to evaluate home literacy/numeracy and TV/tablet exposure in the home environment. In line with previous studies that adopted short questionnaires to evaluate the home environment [11, 42, 53, 79], a four-item questionnaire was adopted so parents could quickly fill it out, encouraging greater adherence to the study. The four questions were referred to: 1) watching TV; 2) watching/playing games on tablets and smartphones; 3) reading/listening to stories with parents; 4) being engaged in activities with numbers (e.g., counting, board games).

The questions were introduced by the sentence: “How often does your child do the following during the week? For approximately how long?”. Responses to the first part of the question (frequency) were on a Likert scale from 1 (never) to 5 (every day). In the second part of the question, parents were asked to rate the time spent on that activity on a typical day of the week; the possible options were: “less than 30 min”, “from 30 to 60 min”, “from 1 to 2 h”, “more than two hours”, scored from 1 to 4 points.

### Children’s Outcomes

*Strengths and Difficulties Questionnaire (parents’ questionnaire).* The single-sided version of the SDQ-parents [94] was administered. This questionnaire includes 25 items describing positive and negative behavioural traits; respondents use a 3-point Likert-type scale (0 = not true, 1 = somewhat true, and 2 = certainly true) to rate each item referring to their son/daughter. The 25 items are divided among the following five scales: Emotional Symptoms (α = 0.67; ω = 0.66), Conduct Problems (α = 0.53; ω = 0.54), Hyperactivity-Inattention (α = 0.70; ω = 0.69), Peer Relationship Problems (α = 0.60; ω = 0.60), Prosocial Behaviour (α = 0.65; ω = 0.65). What is more, a Total Difficulty score (α = 0.78; ω = 0.76) is obtained by adding the scores from the first four scales. A higher score corresponds to more severe difficulties on the four scales describing negative behaviours. On the Prosocial Behaviour scale, a higher score indicates more positive behaviours.

*Literacy, numeracy and cognitive skills (teachers’ questionnaire):* Children’s cognitive and early literacy/numeracy skills and their behavioural profile were assessed with a proxy-report questionnaire administered to their teachers. The items were developed based on the early cognitive, literacy, numeracy, and behavioural skills deemed adequate for preschoolers based on the Italian curriculum for preschoolers and the previous literature. Furthermore, the questions were qualitatively validated by groups of teachers who provided feedback on the items’ clarity. Previous studies found good correlations between teachers’ ratings and objective measures, at least for numeracy [24]. For each item, the name of the competence was accompanied by a short definition and some examples (e.g., *phonological awareness*: “It refers to the child’s ability to perform fusion/segmentation tasks, such as splitting or joining the pieces of the word banana: ba-na-na). The questionnaire consists of 20 items, although three items related to motor coordination were excluded from the present study. The questionnaire included five items on verbal skills (phonological awareness, morphosyntactic comprehension, and production, narrative skills, pre-writing skills); five items on numeracy skills (counting, biunivocal correspondence, cardinality, non-symbolic quantity recognition, number knowledge); three items on cognitive skills (visuospatial working memory, phonological memory, executive functions); and four items on the behavioural profile (ability to respect waiting time, sociality, emotional resources, interest in activities). The teachers rated their evaluations of the children’s skills on a five-point Likert scale from “never/absent” to “always/excellent competence”. The Cronbach’s alpha for the scales is 0.90 (early literacy skills), 0.94 (numeracy), 0.83 (cognitive skills), 0.87 (behavioural profile).

## Procedure

Questionnaires on SES and home literacy/numeracy were provided to parents through paper and pencil questionnaires. Parents could either complete it together or individually by the parent who spends more time with the child, usually the mother. The teachers were required to complete the questionnaire for each child within 1 month in order to allow them to observe the children’s behaviour. The parents of all children involved in the study gave informed consent, and the University of Bologna Bioethical Committee approved the project (Prot. 322,431, December 21, 2021).

## Data Analysis

Descriptives and Pearson correlations were run for all the variables involved.

A structural equation model (SEM; e.g., [100]) was applied using the Mplus software version 7.0 [101]. In this model, two latent dependent variables were identified: Early learning, which includes Verbal Area, Numerical Area, and Cognitive area, and Home Learning Environment, which includes Numeracy time/frequency and Literacy time/frequency. The other dependent variables are the following observed variables: Tablet and TV time, Behavioural Area from the teacher questionnaire, and SDQ total problems from the parents’ questionnaire. This last score was chosen because it was more reliable than the subscales’ scores and because it was more robust in predicting a range of outcomes, compared to the subscales’ scores, in community samples [102, 103]. A path analysis was used to examine the predicting power of SES on the Early Learning, Behavioural area, and SDQ total problems scale through a possible mediation via Home Learning Environment, Tablet and TV time. Gender was included as a control variable. The SEM was run using Maximum Likelihood as the estimator method. To reach a good fit, some adjustments were made following the suggestion of modification indexes without changing the critical structure of the models. Finally, we let the following variables covariate: Tablet with TV time, Numerical Area with Verbal Area, Numeracy time with Literacy frequency, and Numeracy time with Literacy time.

Multiple indices were used to evaluate the models’ fit: Chi-square test of model fit (χ^2^), Root Mean Square Error of Approximation (RMSEA), Comparative Fit Index (CFI), Tucker Lewis Index (TLI). In a non-significant Chi-square test of model fit, TLI and CFI values equal to or higher than 0.90 indicate an acceptable model fit; RMSEA close to 0.08 or lower indicate an acceptable fit [104–106]. Cut-off values for both the RMSEA (0.01, 0.05, 0.08, and 0.10), the CFI/TLI (0.99, 0.95, 0.92, and 0.90) and the Standardized Root Mean Square Residual (SRMR) (< 0.08) have been commonly used to distinguish between excellent, close, fair, and mediocre or poor models, respectively [106].

## Results

Descriptives and gender differences for all the study’s variables are presented in Table 1; Table 2 shows Pearson correlations among them. As for gender differences, some significant results emerged; however, effect sizes for such differences were from null to small [107].

**Table 1:** Descriptives for all the variables of the study and gender differences

		N	Mean	% of children with the highest score	Standard deviation	Min–Max	Skewness (SE = .060)	Kurtosis (SE = .120)	Gender differences
Teacher’s questionnaire	Verbal area	1660	4,02	24.3	0,95	1–5	−0,91	0,11	f > m, *d* = .26
	Numerical area	1660	4,19	35.5	0,91	1–5	−1,15	0,70	f > m, *d* = .14
	Cognitive area	1660	3,85	18.5	0,94	1–5	−0,67	−0,19	f > m, *d* = .32
	Behavioural area	1660	3,92	17.0	0,88	1–5	−0,72	−0,01	f > m, *d* = .49
Parent’s questionnaire	TV time	1658	2,20		0,80	1–5	0,37	−0,17	m > f, *d* = .11
	Tablet time	1654	1,85		0,83	1–5	0,81	0,23	
	Home literacy frequency	1657	3,91		1,22	1–5	−0,61	−1,08	
	Home numeracy frequency	1649	3,36		1,19	1–5	−0,01	−1,18	
	Home literacy time	1631	1,73		0,71	1–5	0,87	1,06	
	Home numeracy time	1580	1,76		0,74	1–4	0,74	0,23	
	Socio-economic status	1660	39,96		13,98	8–70.50	−0,26	−1,22	
	SDQ-Emotional symptoms	1660	1,75		1,81	0–10	1,49	2,79	
	SDQ-Behavioural problems	1660	1,64		1,51	0–8	1,09	1,17	m > f, *d* = .13
	SDQ-ADHD	1660	3,42		2,21	0–10	0,41	−0,45	m > f, *d* = .36
	SDQ-Problems with peers	1660	1,48		1,68	0–10	1,46	2,39	
	SDQ-Prosocial behaviour	1660	7,89		1,77	1–10	−0,89	0,59	f > m, *d* = .30
	SDQ-Total problems	1660	8,29		4,98	0–28	0,97	1,21	m > f, *d* = .26

**Table 2:** Pearson correlations among all the variables of the study

		(2)	(3)	(4)	(5)	(6)	(7)	(8)	(9)	(10)	(11)	(12)	(13)	(14)	(15)	(16)	(17)	(18) Age
Teacher’s questionnaire	(1) Verbal area	0.818^**^	0.808^**^	0.674^**^	−0.082^**^	−0.161^**^	0.202^**^	0.073^**^	0.074^**^	0.023	0.383^**^	−0.083^**^	−0.096^**^	−0.201^**^	−0.233^**^	0.050^*^	−0.227^**^	0.318**
	(2) Numerical area		0.753^**^	0.602^**^	−0.030	−0.138^**^	0.154^**^	0.123^**^	0.064^**^	0.046	0.312^**^	−0.068^**^	−0.086^**^	−0.171^**^	−0.178^**^	0.042	−0.187^**^	0.298**
	(3) Cognitive area			0.762^**^	−0.023	−0.120^**^	0.134^**^	0.075^**^	0.065^**^	0.020	0.292^**^	−0.071^**^	−0.109^**^	−0.220^**^	−0.181^**^	0.040	−0.218^**^	0.244**
	(4) Behavioural area				−0.022	−0.091^**^	0.086^**^	0.046	0.062^*^	0.026	0.178^**^	−0.048	−0.182^**^	−0.262^**^	−0.143^**^	0.102^**^	−0.237^**^	0.237**
Parent’s questionnaire	(5) TV time					0.409^**^	−0.147^**^	−0.079^**^	0.058^*^	0.086^**^	−0.072^**^	0.059^*^	0.088^**^	0.118^**^	0.057^*^	−0.079^**^	0.120^**^	0.040
	(6) Tablet time						−0.201^**^	−0.116^**^	0.027	0.064^*^	−0.165^**^	0.033	0.051^*^	0.110^**^	0.058^*^	−0.066^**^	0.096^**^	0.020
	(7) Home literacy frequency							0.262^**^	0.244^**^	0.021	0.347^**^	−0.055^*^	−0.073^**^	−0.147^**^	−0.102^**^	0.014	−0.142^**^	−0.044
	(8) Home numeracy frequency								0.189^**^	0.301^**^	0.063^*^	−0.042	−0.091^**^	−0.126^**^	−0.032	0.127^**^	−0.110^**^	0.054*
	(9) Home literacy time									0.382^**^	0.089^**^	−0.039	−0.098^**^	−0.167^**^	−0.037	0.128^**^	−0.130^**^	0.033
	(10) Home numeracy time										−0.048	−0.022	−0.074^**^	−0.110^**^	0.012	0.108^**^	−0.075^**^	0.090*
	(11) Socio-economic status											−0.095^**^	−0.057^*^	−0.180^**^	−0.231^**^	−0.093^**^	−0.210^**^	0.030
	(12) SDQ-Emotional symptoms												0.282^**^	0.240^**^	0.507^**^	−0.253^**^	0.727^**^	−0.010
	(13) SDQ-Behavioural problems													0.418^**^	0.178^**^	−0.329^**^	0.651^**^	−0.107**
	(14) SDQ-ADHD														0.174^**^	−0.275^**^	0.716^**^	−0.061*
	(15) SDQ-Problems with peers															−0.267^**^	0.654^**^	−0.108**
	(16) SDQ-Prosocial behaviour																−0.404^**^	0.158**
	(17) SDQ-Total problems																	−0.100**

Notes: ** *p* < .01; * *p* < .05

A paired sample t-test comparing the time spent watching TV and using a tablet revealed that participants spent more time watching TV than using tablets, with a small effect size *t* (1651) = 16.239, *p* < 0.001, *d* = 0.43.

## A Mediation Model of SES on Early Learning and Behaviour

**Fig. 1:**
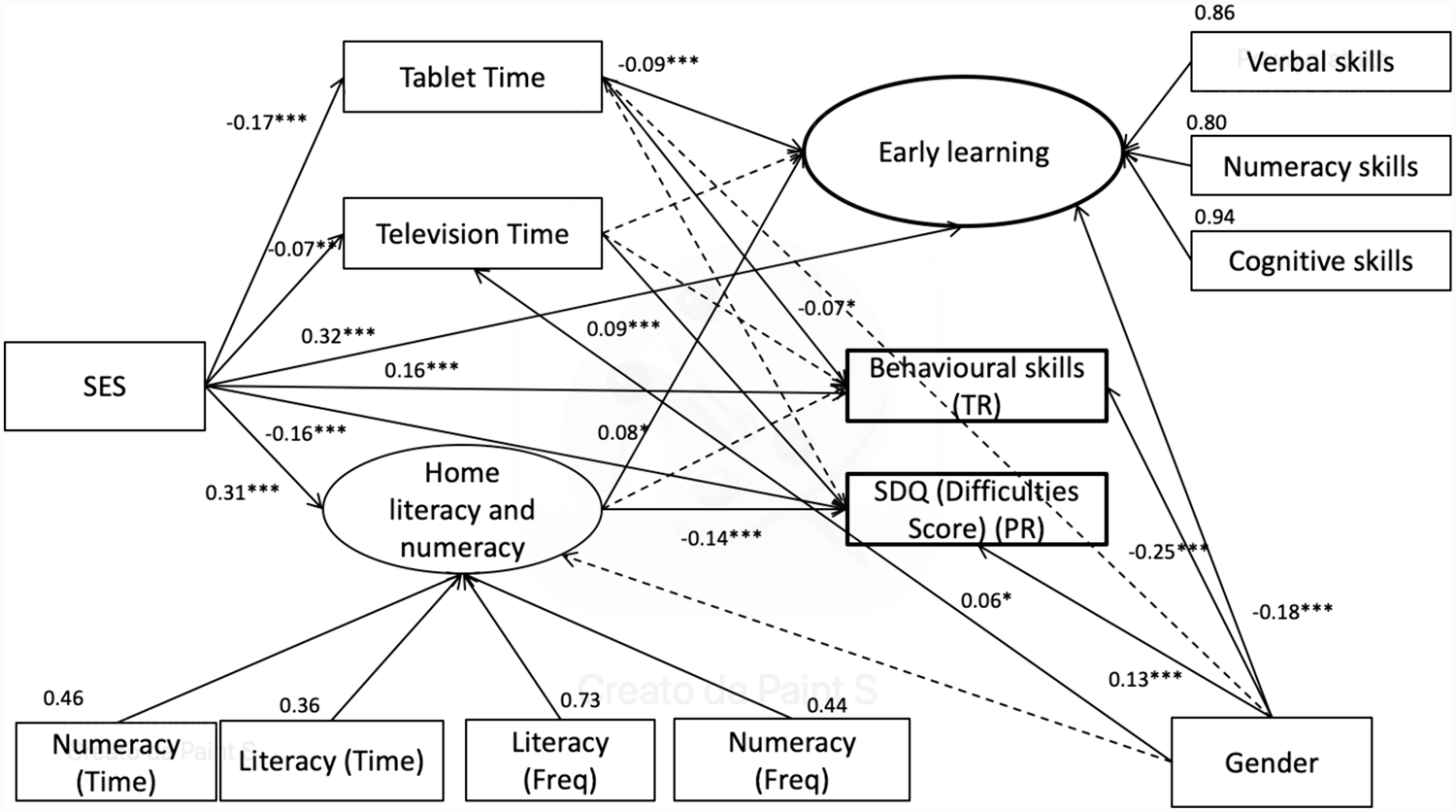
Model depicting concurrent relationships and mediation effects among the variables included in the study

A SEM was performed to better understand the predictive power of SES (Fig. 1), which included home Tablet-TV time and Home Learning Environment as potential mediators.

The SEM’s fit indexes were all acceptable, χ^2^ (42) = 390.488, *p* < 0.001; RMSEA = 0.071; TLI = 0.91, CFI = 0.95, SRMR = 0.044. The hypothesised path from the observed variable (SES) to the latent variables Early Learning and Home Learning Environment was significant (*p* < 0.001); also, a significant path through the observed variables Tablet Time and Behavioural Area (teachers) (*p* < 0.001), Tv Time (*p* < 0.01) and SDQ problems (parents) (*p* < 0.05) was found. The other significant paths were found from our possible mediators and the dependents variable; in particular, Tablet time has a significant negative relationship with Early learning (*p* < 0.001) and with behavioural skills as assessed by teachers (*p* < 0.05), TV time with SQD problems (*p* < 0.001) and finally Home Learning Environment has a significant relation with SDQ problems (*p* < 0.001). Considering gender, being male was negatively related to Early learning and Behavioural skills, and positively related to increased SDQ problems and TV time (modestly).

Then, an Indirect Model was run to test the mediations, and a bootstrap analysis was performed to identify specific indirect relationships in the models. Results showed that Tablet time, decreasing the effect size of this relationship, negatively mediates between SES and Early Learning (*p* = 0.005), with higher SES corresponding to less Tablet time and higher levels of Early Learning. Also, the Home Learning Environment positively mediates the relationship between SES and SDQ problems (*p* = 0.023), with higher SES corresponding to a better home learning environment and fewer behavioural problems. Finally, the Home Learning Environment, increasing the effect size, positively mediates the link between SES and early learning as assessed by teachers (*p* = 0.001), with higher SES leading to a better home learning environment and higher levels of early learning. The same analysis was run also including children’s age as a possible predictor; however, the model’s results were the same, and the model fit worsened. We therefore presented the original model, not including the children’s age as a possible predictor (Table 3).

**Table 3:** Parameters of mediation analysis

*Indirect path*	*β*	*CI lower*	*CI upper*	*p*
*SES—Tablet time—Early learning*	0.015	0.006	0.024	0.005
*SES—Home learning En—Early learning*	0.023	0.006	0.039	0,023
*Ses—Home learning En—SDQ*	−0.041	−0.061	−0.021	0.001

## Discussion

The present study investigated SES’s direct and indirect relationships on early learning and behaviour, using a multi-informant approach (parents and teachers) and involving a large sample of preschoolers. In particular, the home learning environment and the time spent using a tablet or watching TV were the hypothesised mediators of the relationship between SES and the dependent variables, namely early learning skills and behaviour as assessed by teachers and behavioural problems as assessed by parents.

The first aim was to test whether SES predicted home environment practices and children’s learning and behavioural profiles concurrently. It resulted in a significant negative relationship between SES and tablet and television time exposure. On the counterparts, a positive relationship emerged with literacy/numeracy practices. These results reinforce the idea of a direct relationship between SES and family learning practices, with increased screen time for low-SES families [98] and more activities in the literacy/numeracy domain for high-SES families [53, 68]. However, the strength of the association is higher for home literacy/numeracy practices (moderate association) and lower for tablet and TV time (weak association), suggesting high variability and moderate to low effects depending on the specific output considered [70–73]. Considering children’s profiles, in line with previous studies, SES was positively related to early learning skills [51–55] and negatively related to behavioural difficulties as evaluated by parents, suggesting fewer behavioural problems in children from higher SES families [58].

The second aim was to evaluate the relationships between the family environment and children’s early learning and behavioural skills, controlling for gender. It emerged that tablet time, not television time, was negatively related to children’s early learning and behavioural skills, as evaluated by teachers. Furthermore, there was a positive relationship between television time and behavioural problems (parents’ ratings). These results reinforce previous literature on the unfavourable relationship between screen exposure and children’s cognitive and behavioural profiles [7, 84–86]. In particular, the negative outcome of tablet naturalistic use on self-regulation—and particularly emotion regulation [84]—can be the reason for the observed higher scores in behavioural and emotional problems; future studies should directly investigate the role of self-regulation as a mediator between tablet use and behavioural and emotional problems. These results also suggest a more substantial function served by tablets and smartphones compared to television, in line with studies in naturalistic contexts [84] but in contrast with studies that reported better results of interactivity allowed by mobile devices compared to passive viewing. Despite the modality of use of tablets and television, it should be mentioned that tablets are portable devices that can be used in a wider range of environments and situations compared to television. This increased accessibility may lead to prolonged screen time and distractions, potentially exacerbating poor learning and behaviour issues.

In the SEM, two latent variables for early learning skills and behavioural difficulties were considered, which does not allow for the evaluation of relationships with single subscales. However, considering correlation tables, Television Time was negatively related only to the verbal area, whereas Tablet Time was negatively related to all of the teachers’ ratings (verbal, numerical, cognitive and behavioural). Considering the parents’ ratings of behavioural problems, tablet and television time had similar patterns of relationships: the strongest correlations were with problems with peers for both variables. This could be due to the association between television or mobile device use and aggressive behaviours that can affect the relationship between peers [108]; also, time spent in front of screens is likely to reduce playtime with peers [109]. Therefore, we did not reply to the findings reported by [93] indicating a stronger relationship between television time and externalising behaviour.

Finally, the third aim was to test the indirect role of the family’s activities as candidate mediators of the association between SES and children’s learning and behavioural profiles. Two main patterns of results emerged. The first is related to a mediation path of home literacy/numeracy activities on the relationship between SES and children’s early learning skills. These results reinforce findings from previous studies that found mediation effects of home literacy [53] and numeracy [77] practices in the relationship between SES and early learning skills. This suggests that an enriched home learning environment can mitigate the negative relationship between SES and learning prerequisites. Also, the present study has highlighted that the home learning environment mediated the relationship between SES and socioemotional difficulties, as reported by parents. This means a higher involvement in home learning activities can act as a protective factor modulating the relationship between SES and socio-emotional difficulties. As for gender, the impact on the model was small; males emerged with higher scores in social-emotional difficulties, as previously found by literature [95, 112]. Gender differences are not a topic of the present study; however, the impact of gender on the relationship between media usage, environmental variables and learning and behaviour should be considered in future studies.

Finally, tablet time negatively mediated the relationship between SES and early learning skills. The pattern of results, that is, a positive direct relationship between SES and early learning skills and a negative association between SES and tablet time and between tablet time and early learning skills, suggests that spending more time on a tablet might reduce the positive association between SES and early learning skills. A possible explanation of this path can derive from the literature, suggesting that spending time on tablets might act as a distractor, altering parent–child interactions [96, 97], as well as other children’s everyday “real-life experiences” that can support learning.

This study presents some limitations that need to be acknowledged since they might limit the generalisability of results. First, only concurrent predictors were considered, and in the absence of longitudinal studies, proposed paths cannot be interpreted as causal links. Secondly, early learning skills were assessed through teachers’ evaluation, and some critical issues need to be considered. Then, a possible Halo effect—a cognitive bias leading to a homogeneous perception of a person—might have led to the high correlations between the learning and behaviour judgments [111]; indeed, students who behave poorly could be also perceived by teachers as having weaker learning and cognitive skills, and vice-versa. This could partially impact the results of this study, by affecting the independence of the early learning and behavioural factors as rated by teachers. Also, a high percentage of children were rated with the highest scores in literacy and numeracy skills, showing a trend toward a ceiling effect. Despite the skewness values remaining within an acceptable cut-off, this could have partially affected the results, and future studies should use instruments more sensitive to children’s individual differences, particularly at a higher level of learning skills. Furthermore, even though previous research found teacher reports to be reliable and concordant with objective tasks administered to children, a direct evaluation of children’s skills would have given more strength to the study. For example, the teacher’s prejudices against low-SES children could also influence their competence assessment. Then, the SDQ showed weak levels of reliability for some scales (e.g., Conduct Problems), which should be considered when interpreting the results. Finally, the home learning environment was assessed through a short questionnaire, in line with previous studies that adopted short questionnaires to evaluate the home environment [11, 42, 53, 79] to encourage greater adherence to the study. However, as previous literature suggests, this does not allow us to evaluate the distinct effects of home literacy and numeracy skills and those of formal and informal practice. Indeed, previous research has shown the importance of these distinctions in understanding children’s academic development [28]. For example, as for numeracy, formal home numeracy practices (e.g., practising simple sums) have been shown to predict children’s symbolic number system knowledge, whereas informal exposure to numerical games predicts children’s non-symbolic arithmetic [28]. Future studies should utilise more comprehensive measures to explore these factors, enabling a deeper understanding of their unique contributions to different children’s academic outcomes. Also, regarding the evaluation of screen exposure, future studies should address not only the time and frequency of exposure but also the contents and modalities [112], for example, considering who the devices are used with and which types of activities are played. For instance, it has been shown that using mobile technology as a calming tool for upset children particularly affects their self-regulation skills [113]. Also, considering factors such as the passive vs interactive use of each media device, the solitary screen time *vs* screen time as part of social interaction, or analysing the specific content of programs/games, can provide valuable insights into its impact on children’s cognitive, socio-emotional, and behavioural outcomes [83]. Similarly, more efforts should be made to develop HLE models that include the various components suggested by previous literature; in the present study, we did not specifically address the issue of parents’ beliefs and attitudes.

## Summary

The present study reinforces previous findings on the positive relationship between SES and early learning skills and socioemotional well-being and the positive relationships between the home learning environment and early learning skills. Also, it has highlighted a somehow negative relationship between SES and tablet time and between tablet time and early learning skills and socio-emotional well-being. Even if single relationships have been analysed in previous research, the present study adds, as an original contribution, the development of a model that considers the reciprocal interactions among SES, screen exposure, home learning environment, and children’s early learning and behavioural profile. More importantly, this study highlights that the home environment can significantly mediate the relationship between SES and children’s early learning and behavioural profile, either with a positive association with home literacy and numeracy practice or a negative relationship with the time spent using tablets.

The present study offers a new perspective on the reciprocal interactions between SES, the home environment, and children’s cognitive and behavioural outcomes, reinforcing the role of the home environment as an important mediator. Also, the study was conducted on a broad sample and adopted a multi-informant approach involving parents and teachers. Finally, the study was conducted in Italy, where scarce evidence in this regard has been collected so far, and this might pave the way for cross-country comparison studies.

Based on the present findings, some implications for educational programs focusing on the home context might be suggested. Indeed, it appears fundamental to act from a child’s early years to support parents in offering a suited stimulating home environment to their children, particularly for families with low SES. Indeed, this risk factor can be modulated by directly intervening in-home practices, for example, promoting home learning activities and making parents (and educators) more aware of how to expose children to television and tablets.

## Data Availability

The data that supports the findings of this study are available from the corresponding author [PB], upon reasonable request.
